# Breeding for Economically and Environmentally Sustainable Wheat Varieties: An Integrated Approach from Genomics to Selection

**DOI:** 10.3390/biology11010149

**Published:** 2022-01-17

**Authors:** Etienne Paux, Stéphane Lafarge, François Balfourier, Jérémy Derory, Gilles Charmet, Michael Alaux, Geoffrey Perchet, Marion Bondoux, Frédéric Baret, Romain Barillot, Catherine Ravel, Pierre Sourdille, Jacques Le Gouis, on behalf of the BREEDWHEAT Consortium

**Affiliations:** 1 UMR GDEC Genetics, Diversity & Ecophysiology of Cereals, INRAE—Université Clermont-Auvergne, 5, Chemin de Beaulieu, 63000 Clermont-Ferrand, France; etienne.paux@inrae.fr (E.P.); francois.balfourier@inrae.fr (F.B.); gilles.charmet@inrae.fr (G.C.); catherine.ravel@inrae.fr (C.R.); pierre.sourdille@inrae.fr (P.S.); 2 Limagrain, Chappes Research Center, Route d’Ennezat, 63720 Chappes, France; stephane.lafarge@limagrain.com (S.L.); jeremy.derory@limagrain.com (J.D.); 3 Université Paris-Saclay—INRAE, URGI, 78026 Versailles, France; michael.alaux@inrae.fr; 4 Université Paris-Saclay—INRAE, BioinfOmics, Plant Bioinformatics Facility, 78026 Versailles, France; 5 Vegepolys Valley, Maison du Végétal, 26 Rue Jean Dixmeras, 49066 Angers, France; geoffrey.perchet@vegepolys-valley.eu; 6 INRAE—Transfert, 5, Chemin de Beaulieu, 63000 Clermont-Ferrand, France; marion.bondoux@inrae.fr; 7 UMR EMMAH, INRAE—Université d’Avignon et des Pays de Vaucluse, 84914 Avignon, France; frederic.baret@inrae.fr; 8 INRAE, URP3F, 86600 Lusignan, France; romain.barillot@inrae.fr

**Keywords:** wheat, *Triticum aestivum*, wheat breeding, molecular tools, genomic selection, high throughput phenotyping, diversity, wheat database

## Abstract

**Simple Summary:**

The BREEDWHEAT project has laid the foundation for future commercial varieties by providing new and original modern molecular tools to breeders who have applied them to: (1) efficiently analyze and structure the genetic diversity; (2) decipher traits of agronomical interest including biotic and abiotic resistance and tolerance; (3) develop methodologies to implement genomic and phenomic selection; (4) store and bring tools to the world wheat community to access all of the results and data. This will provide a helping hand to developing new original and adapted wheat varieties that will be more productive in terms of the quantity and quality in the context of sustainable agriculture using less fertilizers, pesticides, fungicides, and water. This would help in tackling the challenges that we have to face, especially with regard to global change.

**Abstract:**

There is currently a strong societal demand for sustainability, quality, and safety in bread wheat production. To address these challenges, new and innovative knowledge, resources, tools, and methods to facilitate breeding are needed. This starts with the development of high throughput genomic tools including single nucleotide polymorphism (SNP) arrays, high density molecular marker maps, and full genome sequences. Such powerful tools are essential to perform genome-wide association studies (GWAS), to implement genomic and phenomic selection, and to characterize the worldwide diversity. This is also useful to breeders to broaden the genetic basis of elite varieties through the introduction of novel sources of genetic diversity. Improvement in varieties particularly relies on the detection of genomic regions involved in agronomical traits including tolerance to biotic (diseases and pests) and abiotic (drought, nutrient deficiency, high temperature) stresses. When enough resolution is achieved, this can result in the identification of candidate genes that could further be characterized to identify relevant alleles. Breeding must also now be approached through in silico modeling to simulate plant development, investigate genotype × environment interactions, and introduce marker–trait linkage information in the models to better implement genomic selection. Breeders must be aware of new developments and the information must be made available to the world wheat community to develop new high-yielding varieties that can meet the challenge of higher wheat production in a sustainable and fluctuating agricultural context. In this review, we compiled all knowledge and tools produced during the BREEDWHEAT project to show how they may contribute to face this challenge in the coming years.

## 1. Introduction: Wheat in a Fluctuating World

**Figure 1:**
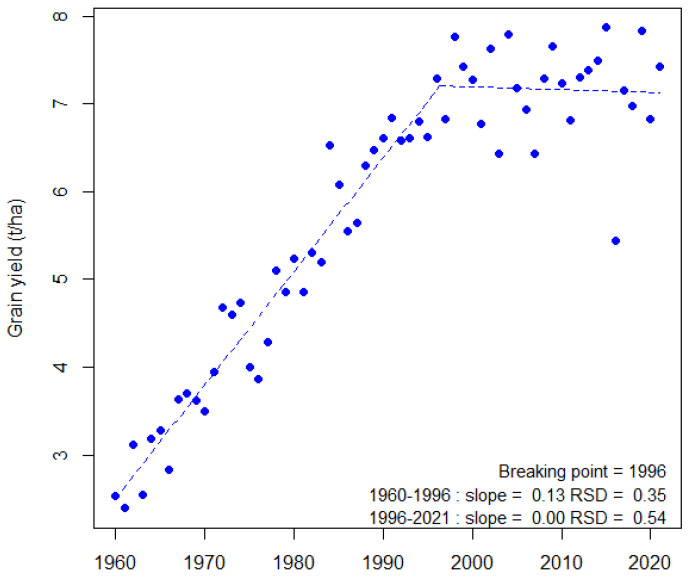
Evolution of bread wheat grain yield from 1960 to 2021 in France. A bi-linear regression model was fitted (blue dotted line) for grain yield using the segmented R package [7] with default settings. The value of the breaking point and the values of slope and standard deviation of the residuals (RSD) are indicated for each period. Data were from the Agreste database (https://agreste.agriculture.gouv.fr/agreste-web/accueil/ (accessed on 31 October 2021)).

With 220 million hectares leading to an annual production of 729 million tons, bread wheat (*Triticum aestivum* L.) is one of the most important crops worldwide and the staple food for one third of the world’s population. It is also a major renewable resource for feed and raw industrial materials. With changing diets and growing world populations, rising prices for fertilizers and phytosanitary products, and an increasing competition between food and non-food uses, the demand is continuously growing. Thus, today’s agriculture has to face an unprecedented challenge: to keep pace with the human demand in an environmentally and socially sustainable manner [1]. To meet this challenge, wheat yield should increase by 1.7% per year over the next 30 years while the current yield increase worldwide is only 0.9% per year, stagnating even in the main producing countries [2]. This goal would be achievable under the assumption of favorable growing conditions but is more unlikely under climate change, which affects not only yield but also yield stability [3,4,5]. With its high yielding wheat production (7.2 t ha-1 average on 2017–2020 in France), the EU28 is the first ranked world wheat producer (148 Mt in 2019) and therefore its production contributes significantly (22%) to the world supply. France alone ranks fifth (35 Mt year-1 average on 2017–2020) in the world and first in the EU for both production and export. However, like in many other countries, French annual yields have been stagnating and are highly volatile since the end of the 1990s (Figure 1) [3,6].

Thus, there is a need to speed up genetic progress for yield potential as well as to improve tolerance to biotic and abiotic stresses, which are expected to increase in frequency and intensity as a consequence of climate change. New resources and methods that will help deliver improved varieties within shorter selection cycles (e.g., doubled-haploid, marker-based selection, speed breeding, genomics selection, genome editing) are therefore needed.

## 2. The BREEDWHEAT Project Addressed Several Issues

In a large public-private partnership, the BREEDWHEAT project was designed to address the aforementioned challenges through the development of knowledge, resources, tools, and methods that could accelerate the translation from genomics to breeding through a combination of genomics, genetics, ecophysiology, modeling, and phenotyping analyses. BREEDWHEAT targeted the identification of QTLs and genes underlying key traits for adaptation to abiotic and biotic stresses to enable breeding programs to better exploit genetic resources and adapted germplasm through innovative breeding and management methodologies. From 2011 to 2020, BREEDWHEAT brought together 26 public and private research groups (https://breedwheat.fr/partners/?lang=en (accessed on 16 December 2021)) with extensive complementary skills and resources. It represented a unique opportunity to integrate and synergize the efforts developed so far in small-scale and short-term projects.

To reach its objectives, BREEDWHEAT aimed at facilitating the translation of knowledge and molecular resources into breeding as well as better exploiting genetic resources to enlarge the diversity of the wheat gene pool.

Genome sequences hold the key for understanding the molecular basis of phenotypic traits variations and provide a framework for varietal development through the utilization of marker-assisted selection. Despite the recognition that genome sequencing is critical for crop improvement, the size (approx. 16 Gb, 5× the human genome and 40× that of rice), and complexity (three homoeologous A-, B-, and D-genomes and more than 80% of repetitive DNA) of the wheat genome have long been obstacles to the efficient development of genome sequencing projects. In 2009, when the BREEDWHEAT project was devised, wheat was the last major crop for which no reference genome sequence was available. The physical map of the largest chromosome, 3B, only just published [8], established a template for the remaining wheat chromosomes and demonstrated the feasibility of constructing physical maps in large, complex, polyploid genomes with a chromosome-based approach. On this basis, BREEDWHEAT teamed up with the International Wheat Sequencing Consortium (IWGSC) to provide the wheat community with this resource.

The elite crops used in modern agriculture have been developed through domestication and selection by farmers and plant breeders over hundreds, sometimes thousands of years. The genetic variability of the elite germplasm has decreased as a consequence of reduced population size (also called “bottleneck”) due to domestication, genetic drift, and modern selection. While the frequency of the utmost adapted alleles is increasing, many others, nearly neutral but potentially adaptive, have been lost [9,10]. It has become increasingly clear that the commonly used elite genetic pool will hardly enable the genetic gain needed for yield and quality in the context of global change. Thus, it is necessary to more efficiently exploit worldwide regional landraces, local varieties as well as wild relatives that likely contain novel and unique alleles that will sustain innovation in breeding [11]. BREEDWHEAT aimed at contributing to an in-depth characterization of the worldwide bread wheat genetic diversity and establishing new pre-breeding populations and panels.

To ensure global food security, wheat grain yield (GY) needs to be increased in the context of global change and the concomitant reduction in the use of water, fertilizers, and pesticides due to environmental issues [1,12]. BREEDWHEAT aimed at addressing these issues by identifying original genitors from exotic or ancient genetic resources and relevant agronomical traits, molecular processes, genes, and loci associated with response to low nitrogen input, high temperature, drought, and major fungal diseases that will constitute the major challenges to face in the coming years.

More than 80% of world wheat production is used after industrial processing, which requires specific protein concentration and composition. Both grain protein concentrations (GPC) and storage protein composition are key to the technological quality. These are dependent on nitrogen (N) and sulfur (S) availability for the crop. It is likely that the predicted reduction in N fertilizer use and the continuous reduction in anthropogenic S deposition will alter the dough characteristics. The synthesis of grain storage protein is primarily regulated at the transcriptional level by a transcriptional complex [13]. A better understanding of the regulatory networks controlling the expression of genes for grain storage proteins holds the key to engineer flour characteristics. BREEDWHEAT aimed at contributing to the identification of key actors (e.g., transcriptional factors) involved in the grain response to N and S supplies that can be used to maintain the bread making quality of new cultivars in the context of sustainable agriculture.

To reach these objectives, BREEDWHEAT also addressed a number of methodological and technological challenges including high-throughput genotyping and phenotyping as well as genomic selection. In this review, we will provide an overview of the main achievements of the project and show how they could be useful for wheat breeders worldwide.

## 3. A Genomics Toolbox for Wheat Research and Breeding

Genomics tools have the potential to assist conventional breeding to develop better varieties more rapidly through genome-wide association studies, characterization of genetic resources, marker-assisted breeding (including genomic selection), genome editing, etc. However, because of its size and complexity (allohexaploid and highly repetitive), the bread wheat genome has long been perceived as too complex for the efficient development of genetic and genomic resources such as highly saturated genetic maps and a reference genome sequence. Consequently, wheat has long lagged behind other crops in terms of molecular-assisted breeding and the characterization of genes underlying major agronomic traits. BREEDWHEAT participated in the development of several genomics tools that are summarized in Table 1 and are detailed in the following sections.

### 3.1. Polymorphism Detection and High Throughput Genotyping

Single nucleotide polymorphisms (SNPs) have been adopted as the markers of choice for genetic studies because they are the most abundant type of polymorphism in plant genomes and because they are amenable to high-throughput, cost effective genotyping technologies [27,28]. In 2011, at the beginning of the BREEDWHEAT project, only a limited number of SNPs were published in wheat and no high-throughput technologies were available. The development of the BREEDWHEAT TaBW280K Axiom SNP array was initiated in 2012, simultaneously with the Axiom 820K chip [29] and finalized in 2013, concomitantly with the publication of the first SNP array in wheat [30]. This array took advantage of pre-publication access to the IWGSC chromosome-based draft assembly of the wheat genome [31]. By combining these sequences with resequencing data from eight wheat cultivars, BREEDWHEAT discovered more than three million SNPs and selected a subset of 280,226 polymorphisms located in both genic and non-repetitive intergenic regions to build an Affymetrix Axiom array [14].

This array was complemented with additional SNPs coming from an ISBP (insertion site-based polymorphism) capture experiment. To this aim, the technique later reported by Cubizolles and collaborators [32] was applied to 96 wheat accessions comprising 13 elite varieties and 83 accessions selected to represent the worldwide genetic diversity. Eventually, 350 k SNPs were identified and a subset of 105,703 was selected that were polymorphic in elite material and with low rates of missing and heterozygous data. In addition, 4815 SNPs brought by BREEDWHEAT partners, 116 published polymorphisms in major genes (*Rht*, *Vrn*, and *Ppd*) as well as 5155 SNPs from the Axiom 820K [29] and 13,670 SNPs from the Illumina 90K SNP arrays [33] were added. Altogether, 409,685 polymorphisms were incorporated in the BW SNP chip (called TaBW410K SNP array).

In 2017, a subset of this array comprising 34,746 SNPs (therefore called TaBW35K SNP array) was selected using several complementary criteria in order to maximize its usefulness. We chose polymorphic high resolution or off-target variant SNPs located on all wheat chromosomes and with high quality clustering profiles [22]. These SNPs were evenly distributed based on linkage disequilibrium analyses (r^2^ = 0.80), and preferably associated with agronomic traits. In addition, when possible, the priority was given to SNPs that were shared with the Wheat Breeder’s Axiom 35K [34] and the Illumina 90K SNP arrays [33] to allow for comparative analyses of panels genotyped with these two latter chips and the two TaBW410K and TaBW35K SNP chips.

The TaBW410K and TaBW35K SNP arrays were proven to be highly useful for the BREEDWHEAT project (see following sections) and Axiom arrays were more powerful than Illumina arrays that required a lot of manual curation of the data [30,33].

### 3.2. Genetic Mapping and Recombination Pattern Analyses

Analysis of complex polygenic traits relies on the establishment of densely populated genetic linkage maps of molecular markers [35]. Using the TaBW410K SNP array [15] on 430 F6 recombinant inbred lines (RILs) derived from the cross between Chinese Spring and Renan (CSRe population), we were able to genetically map 146,602 SNPs on the 21 bread wheat chromosomes. This was the largest set of mapped markers used to anchor and order the scaffolds and elaborate the 21 pseudomolecules of the wheat genome sequence [22]. This map confirmed that recombination mainly occurs in distal chromosomal regions favoring allelic diversity, providing the basis for adaptability to changing environments. This study suggested that recombination could be one of the main drivers of the partitioning of the chromosomes that is observed in wheat with two highly (R1 and R3) and two poorly (R2a and R2b) recombinogenic regions [36].

Such a high-density of markers allows for the application of different approaches to study the recombination rate, especially those based on the variation of linkage disequilibrium (LD) or coalescent analysis [37]. By using the genotyping data from the TaBW410K SNP array and densifying some regions of chromosome 3B, it was shown that crossovers (COs) derived from the CSRe population correlated quite well with ancestral COs (predicted through linkage disequilibrium) obtained from two collections of 180 varieties representative of the Asian and European genetic pools [38]. Moreover, the high density of SNPs allowed us to observe a significant association of COs with genic features as well as a higher frequency of a DNA motif specific to the TIR-Mariner DNA transposon in recombinant intervals. A similar approach was adopted using the same SNP array to genotype 371 landraces representing four diverging populations (West Asia, East Asia, West Europe, and East Europe) [39]. Recombination landscapes of the four populations positively correlated between each other and significantly shared most recombinogenic intervals. Interestingly, correlation was the highest for the less divergent populations, suggesting an effect of SNP divergence on recombination rates. Finally, 118,189 SNPs of TaBW410K SNP array with no missing data were used to estimate the pairwise distances between 29 varieties used to develop a nested associated-mapping (NAM) population [40]. This population was developed using the CIMMYT photoperiod insensitive cultivar “Berkut” as the pivotal parent crossed with 28 lines, resulting in 2100 F6 recombinant inbred lines (RILs; 75 individuals per population) and was also used to detect QTLs for grain protein content [41,42,43]. These data, combined with genotyping by sequencing (GBS) data of segregating individuals, led to the location of quantitative trait loci (QTLs) affecting the frequency of COs. Some of these QTLs contained candidate genes known to be involved in recombination variation such as Hei10 on chromosomes 6A and 6B.

Dense genetic maps also greatly help in positional cloning of genes of interest. For example, 84 SNPs from the TaBW410K array were used to characterize a set of 113 deletion lines from chromosome 3D [44]. The size of the deletions ranged from 6.5 to 357.0 Mb but most interestingly, the regions between two successive deletions (deletion bins) ranged from 0.15 to 50.00 Mb, therefore reducing the number of genes in each bin. This was a crucial step toward the positional cloning of *Ph2*, a gene controlling homoeologous recombination in wheat [45]. With these SNPs, we revealed that the initial deletion in the *ph2a* mutant in fact covered 121 Mb and not 80 Mb, as initially estimated. Most importantly, we reduced the region bearing *Ph2* to a segment of 14.3 Mb containing only 100 genes, *TaMsh7-3D* being the only relevant candidate.

### 3.3. Sequencing the Bread Wheat Genome

To overcome the difficulties related to the size and complexity of the bread wheat genome, the IWGSC decided to adopt a chromosome-specific approach to construct integrated physical maps and sequence the hexaploid wheat genome [46]. In 2011, this strategy had been successfully applied to chromosome 3B and had led to the publication of the first physical map of a wheat chromosome, 3B [8]. This also resulted in the first project funded at the international level for the production of a high-quality reference sequence of a wheat chromosome [36]. Other physical maps have already been constructed, though not published as yet. This included the chromosome 1BS and 1BL physical maps that were constructed in the framework of the EU FP7 TriticeaeGenome project [47] and published in 2013 [48,49].

With the aim of contributing to the international effort to sequence the hexaploid wheat genome, BREEDWHEAT sequenced chromosome 1B in collaboration with France Genomique (www.france-genomique.org/platforms-and-equipments/ (accessed on 16 December 2021)). A total of 10,395 BAC clones corresponding to the physical map minimal tiling path (MTP) was selected. Pools of ten BACs were sequenced using an Illumina MiSeq in 2 × 250-bp overlapping pairs. Pools of 96 BACs were sequenced in 2 × 100-bp 5-kb mate-paired reads using an Illumina HiSeq2000. The assembly was conducted with the Newbler and SSPACE algorithms [50,51], leading to 13,227 scaffolds with an N50 of 351 kb and a cumulative length of 920 Mb [22]. In parallel, BREEDWHEAT also contributed to the construction of other physical maps such as that of chromosome 7DS by providing genetic mapping data of the Chinese Spring × Renan population for physical contig anchoring and ordering [52].

The IWGSC reference sequence led to unprecedented analyses of the wheat genome. To date, the IWGSC reference sequence has been cited in more than 650 articles in different fields including the transcriptional landscape of wheat and the expression and regulation of homoeologous genes [23,24], the impact of transposable elements on genome structure and evolution, etc. [25].

## 4. Characterization and Exploitation of the Wheat Genetic Diversity

From an historical point of view, bread wheat is an allohexaploid species arising from two hybridization events between three diploid species: *Triticum urartu* (A-genome), an *Aegilops* species from the *Sitopsis* section (S-genome), and *Aegilops tauschii* (D-genome) [53,54]. During the processes of domestication, wheat has undergone important genetic bottlenecks, resulting in a very narrow genetic base, especially when considering the D-genome [55]. More recently, modern breeding has also participated in reducing the genetic variability, especially in winter elite germplasm [11]. The introduction of novel sources of genetic diversity within the elite germplasm is one of the most promising approaches for genetic improvement of the cultivated varieties. Novel sources of diversity are present in different types of material. The most obvious correspond to cultivars that are grown in various agro-climatic environments around the world, in particular, cultivars that are adapted to very stressful environments (e.g., under dry, hot, and/or with high disease pressure). A second source is the large amount of diversity present in so-called Genebanks, which corresponds to several thousands of genotypes that are kept alive in collections [56].

### 4.1. Characterizing the Worldwide Genetic Diversity

With 11,960 hexaploid wheat accessions originating from 108 countries, the INRAE Biological Resource Center (www6.clermont.inrae.fr/umr1095/crb (accessed on 16 December 2021)) is one of the largest ex situ collections in the world. It is composed of 32.5% French cultivars, 27% European cultivars, and 40.5% cultivars from the rest of the world. Inside this collection, different biological classes are represented such as landraces and traditional cultivars (20%), modern varieties (36%), and breeding material (44%). Significant efforts have already been made to describe and characterize this collection. A first core collection covering more than 98% of the diversity (based on neutral markers) of the entire collection was developed [57] and described for both phenotypic traits [58,59] and molecular data [60].

**Figure 2:**
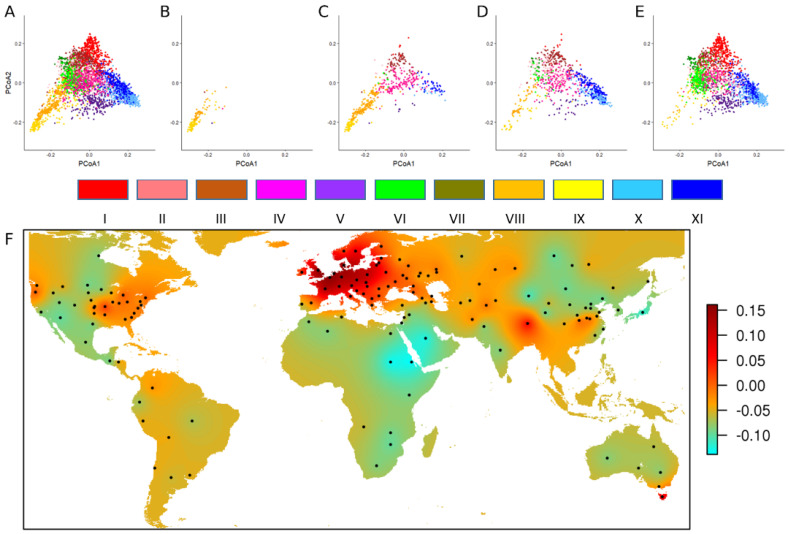
Temporal evolution of worldwide genetic diversity. PCoA calculated with 8741 haplotypes (**A**) on 4403 accessions, (**B**) on 139 Southeast Asia and Indian Peninsula landraces, (**C**) on 632 landraces, (**D**) on 947 traditional cultivars, and (**E**) on 2210 modern varieties. The different colors correspond to the 11 groups defined with a phylogenetic analysis [16]. Group I: modern lines, Southeast Europe; Group II: Northwest Europe, North America; Group III: North America, Southeast Europe; Group IV: Northwest Mediterranean; Group V: modern lines, China, Italy; Group VI: modern lines, CIMMYT, Northwest China; Group VII: modern lines, Canada, China, USA; Group VIII: Southeast Asia, India, Pakistan; Group IX: China, Japan; Group X: modern lines, France, UK; Group XI: France, Germany. (**F**) Geographical projection of the first axis of the PCoA for 2210 modern varieties.

To better characterize the whole collection, 4506 accessions were selected based on sampling an optimal worldwide diversity using the available passport data including geographical origin (country, region, state, department…), status (landraces, breeding lines, cultivars, elite lines…) registration period, growth habit (spring, intermediate or winter type), and pedigree. This panel was genotyped using the TaBW280K SNP array and a worldwide phylo-geographical study was conducted to trace back the history of the wheat genetic diversity (Figure 2) [16].

### 4.2. Assembling a New Pre-Breeding Panel for the European Breeding Programs

At the beginning of the BREEDWHEAT project, an elite panel (BWP2 panel) was assembled to represent the diversity of cultivated winter wheat in France. The panel was composed of 220 European elite varieties of winter wheat released by the different breeders between the mid-1970s and the early 2010s, 89% of which have been mostly released in France since 2000. This panel was extensively used for genome wide association studies on several agronomic traits (see below, [17,18,19,20,21,61]).

**Figure 3:**
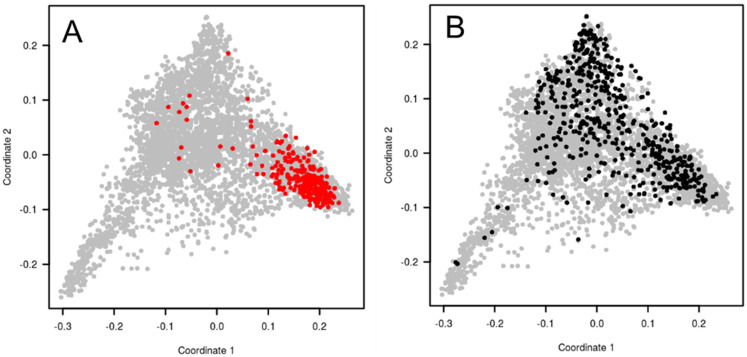
Principal coordinate analysis (PCoA) of bread wheat accessions calculated with TaBW410 SNP markers. (**A**) in grey, 4506 worldwide accessions of the INRAE Biological Resource Center, in red, 220 elite European winter varieties (BWP2 panel). (**B**) in grey, 4506 worldwide accessions of the INRAE Biological Resource Center, in black, a sub-sample of 450 winter wheat accessions selected based on plant height, heading date, and SNP markers, designed for association studies (BWP3 panel).

It was decided to select a complementary winter wheat pre-breeding panel representing the worldwide diversity and optimized for association genetics. Out of the 4506 accessions previously characterized, 1340 accessions were referenced as winter type on passport data and were adapted to French growing conditions (based on comparison to standard varieties) regarding plant height and heading dates. The objective was to design a panel of about 500 accessions for association studies. Two sampling strategies were investigated with the aim to minimize future spurious associations due to structure, and as far as possible with a limited allelic diversity reduction. Both the maximum length sub-tree (MLS) sampling strategy and minimal SD subset (MSDS) sampling strategy were implemented in DARwin software [62]. The MSDS sample was finally chosen based on lower global LD values and more diverse geographical origins of the accessions. Indeed, the MSDS strategy tries to find a sub-sample that shows the smallest disequilibrium. The procedure is a stepwise algorithm, removing at each step the unit with the greatest contribution to the general disequilibrium between all pairs of loci. This new panel (BWP3 panel) sampled a much larger diversity than the elite panel, as shown with the principal coordinate analysis based on the TaBW280K SNP array (Figure 3). Finally, set to a size of 450 accessions, this panel was consequently used for association studies. The list of the accessions and seeds are available upon request to the INRAE Biological Resource Center.

## 5. Genetics and Ecophysiology Studies of Wheat Adaptation to Biotic and Abiotic Stress in the Framework of Sustainable Agricultural Systems and Climate Change

The grain yield increase that is needed in the next few decades has to be achieved in the context of climate change with increased atmospheric CO_2_ levels. This may be favorable to C3 plants in the first place [63]. However, the increasing frequency and intensity of high temperatures and water deficits that will accompany climate change will eventually negatively impact grain yield as well as grain weight and protein composition, two major determinants of wheat end-use value [3,64,65,66]. In addition, the reduction in nitrogen (N) fertilization and fungicide application planned in keeping with the framework of sustainable agriculture will also potentially affect grain yield and protein quality [67]. Sulfur (S) availability, which impacts wheat yield and end-use value, will most likely also become a concern in the coming years because of the tremendous reduction in atmospheric deposition since the 1970s [68,69]. Thus, breeding for new varieties adapted to these constraints, in other words, with improved tolerance to major abiotic (limited water supply, high temperature, limited N and S availability) and biotic stresses (fusarium head blight, septoria leaf blotch), and able to efficiently exploit higher atmospheric CO_2_ concentration is of critical importance [5]. Identifying traits and markers associated with a better tolerance to these major environmental factors will help to select such varieties.

### 5.1. Grain Composition

Improving yield potential while maintaining grain quality is a huge challenge, especially in the sustainable agriculture context, which implies to decrease nitrogen inputs. The sulfur deficiency recently observed in soils adds to this context. Both N and S are essential for grain storage protein (GSP) synthesis and then for grain quality. Thus, identification of the molecular mechanisms that control the accumulation of GSP in response to N and S supply is necessary to maintain/improve cereal grain nutritional and functional properties. To reach this goal, we developed a large-scale analysis to characterize the grain response to N and S deficiencies as well as a study focusing on transcriptional factors involved in GSP synthesis.

For the large-scale analysis of characterizing grain response to N and S deficiencies, an experiment based on a genotype of einkorn (*Triticum monococcum* ssp. *monococcum*) was performed. Einkorn was used as a good diploid model species to study bread wheat GSP regulations. It was cultivated in an environmentally controlled growth chamber as described in Bonnot et al. [70]. Plants received a nutrient solution containing both N and S, which was replaced at anthesis with demineralized water. Then, during grain filling (from 200° Cd to 700° Cd after anthesis), four combinations of N and S were supplied: N−S−, N+S−, N−S+, N+S+, according to whether N and/or S were applied. Samples of grains were harvested at different thermal times after anthesis to provide proteomic, transcriptomic, and metabolomic data. In particular, the proteomic approach focused on GSP, albumins-globulins as well as nuclear proteins. Studying the nuclear proteome was possible thanks to the development made by Bancel et al. [71]. To see how grain proteome, transcriptome and metabolome responded to different amounts of N and S during grain development, we first analyzed the proteomic-data separately. Then, a large-scale analysis including all omics-data was performed. Data integration was based on a network approach using either RulNet (https://wheat-urgi.versailles.inrae.fr/Tools/RulNet (accessed on 16 December 2021)), a web-oriented platform partly funded by BREEDWHEAT [72], or Mixomics (http://mixomics.org/ (accessed on 16 December 2021)).

As reported by Bonnot et al. [70], the N to S ratio in the grain was clearly affected by post-flowering N and S nutrition. This led to major changes in the grain proteome due to perturbation in the N:S balance. This changed the GSP composition in mature grain, by modification of the rate and duration of GSP accumulation. Post flowering N and S nutrition also influenced grain nuclear and albumin-globulin fractions, as shown by the differentially abundance of 203 proteins from these fractions. S supply strongly increased the rate of accumulation of S-rich α/β-gliadin and γ-gliadin, and the abundance of several other proteins involved in glutathione metabolism. Post-anthesis N supply resulted in increasing high molecular weight glutenins and ω1-2 gliadins. It also activated the amino acid metabolism at the expense of carbohydrate metabolism and the activation of transport processes including nucleocytoplasmic transit. Protein accumulation networks obtained due to RulNet emphasized the strong importance of S. The abundance of DNA-binding proteins was modified by the treatments, suggesting a transcriptional reprogramming with potential effects on chromatin. This detailed analysis of grain sub-proteomes provides information on how wheat grain storage protein composition can be managed in low-level fertilization conditions. To complete this work, the response to N and S nutrition of albumin-globulin proteome of the grain in development was re-analyzed to establish relationships between the storage proteins and all quantified and identified the albumins-globulins (*n* = 352) present in all samples [73]. Both datasets were integrated using methods implemented in Mixomics to find candidate albumins-globulins related to seed storage synthesis. This approach was completed by linkage mapping to identify the candidate albumins-globulins statistically associated with storage proteins. These integrative approaches highlighted 18 albumins-globulins, out of which three were statistically validated by association genetics. Four out of these 18 proteins were also highlighted by Bonnot et al. [70], two of them being associated with storage proteins.

The final step of this work concerned a large-scale analysis including all omics-data to identify regulatory mechanisms that may be involved in the control of grain composition in response to N and S nutrition [74]. Twenty-four transcripts were identified as potential coordinators of the grain response to N and S supply and strongly responded to S deficiency. They emphasized the high impact of S deficiency on the transcriptome and metabolome of developing einkorn grains. Post-anthesis N supply without S increased the pool of free amino acids, necessary for GSP synthesis. In response to the increase in the grain N-to-S ratio and the resulting grain S deficiency, sulfate transporters and genes involved in methionine metabolism were upregulated, suggesting regulation of the pool of free amino acids and of the grain N-to-S ratio to probably limit the impact of S deficiency.

For the study focusing on transcriptional factors involved in GSP synthesis, we identified a regulatory protein called SPA heterodimerizing protein (SHP) by looking for orthologs of transcription factors involved in storage protein synthesis in barley [75]. SHP is encoded by three homoeologous genes located on group 5 chromosomes. It bounds cis-motifs of the promoters of high and low molecular weight glutenin genes that were already reported to bind to bZIP family transcriptional factors. Contrary to its barley ortholog, it acts as a repressor of their activity. This result was confirmed by transgenic lines. Two SHP over-expressed events were available. These lines and their null segregant lines were cultivated with low and high nitrogen supply. SHP relative expression at 500 °C days after anthesis was five- and eight-fold higher in the overexpressed lines compared with the null segregant lines in both N treatments. Their phenotype showed a lower quantity of high and low molecular weight glutenins, while the quantity of gliadin did not change, regardless of the availability of N. This led to an increase in the gliadin to glutenin ratio, suggesting differences in the regulation of gliadin and glutenin genes.

Taken together, these studies point to several genes or proteins involved in the adaptation of grain protein composition to nutritional deficiencies. Although several candidate genes were identified, they showed that the regulation of storage proteins is complex and needs to be further investigated.

### 5.2. Adaptation to Abiotic Stress

#### 5.2.1. Heat Stress

Wheat is sensitive to high temperature during its reproductive phase, particularly, a few days before and after anthesis [76]. Depending on their timing, intensity, and duration, high temperatures can reduce either the number of grains per ear [77], or the final grain dry mass [76], or both. Alterations in grain development result from various molecular and cellular responses at different levels that have been widely addressed in the literature. However, they were mainly investigated after a severe heat stress in the leaves. In contrast, the effect of a high, but relevant in Northern European growing conditions, temperature on grain development is less documented in wheat. In this context, within BREEDWHEAT, Girousse et al. [78] first analyzed the response of wheat grain development in response to low and moderately high temperatures (19 °C vs. 27 °C) in two genotypes. They used the NimbleGen microarray containing 40,656 UniGenes to characterize differentially expressed genes. In these conditions, grain dry mass was reduced by 14.4% and 6258 genes had an expression level affected by the temperature at one or more of the developmental stages. Both the up- and downregulated genes were then annotated and their enrichment, in particular, metabolic pathways was investigated. The upregulated genes were particularly enriched in genes involved in “nutrient reservoir activity”. This suggests that moderately high temperatures induce an earlier expression of genes related to nutrient accumulation. This process may be the signal for an accelerated development rate of the grain, which results in a net reduction in grain dry mass. Then, Touzy et al. [20] explored, under the same conditions, the genetic variability for tolerance to heat in the panel of elite Northern European winter varieties (BWP2 panel) previously genotyped [14]. They identified a significant variability for the tolerance, with grain dry mass losses at high temperature ranging from 9.1% to 36.4%. A GWAS was carried out with TaBW410K to dissect the genetic determinants of heat tolerance. Ten QTLs were associated with at least one trait and seven QTLs were characterized by a significant interaction with post-anthesis heat stress. In particular, a significant SNP × treatment interaction for grain dry mass was identified on the telomeric region of the short arm of chromosome 4B. Focusing on a well-defined moderate terminal heat stress, these findings will help identify the genomic regions needed to develop heat-stress tolerant crops.

#### 5.2.2. Drought Stress

Drought is one of the main abiotic stresses limiting winter bread wheat growth and productivity. Alleles for tolerance in one drought scenario could have negative effects under other growing conditions, generating genotype × environment (G×E) interactions [79]. Defining the relevant target water stress scenario is then the first necessary step. Quantifying the genetic variability and identifying chromosomal regions involved is the second step. Several genetic studies have already been conducted and QTL reviewed [80,81,82]. To our knowledge, however, no study has explored the differences in water stress scenarios in a multi-environment trial of European winter wheats. The same panel of elite Northern European winter varieties (BWP2 panel) as used in Touzy et al. [20] was experimented on by BREEDWHEAT partners in 35 field trials [21]. A crop model was run with detailed climatic and soil data to assess the dynamics of water stress in each environment. These simulations allowed for grouping the environments into four water stress scenarios: an optimal condition with no water stress, a post-anthesis water stress, a moderate water stress around anthesis, and a high pre-anthesis water stress. Interestingly, a significant genetic progress was observed for both the optimal condition and the high-stress scenarios. The GWAS identified several QTLs, some of which were specific to the different water stress patterns. These results make easier breeding for improved drought resistance to specific environmental scenarios. This will facilitate genetic progress for future environmental conditions (i.e., water stress environments).

#### 5.2.3. Nitrogen Stress

N is a major plant nutrient whose application strongly increases grain yield and grain protein concentration. There are, however, many adverse environmental effects of inappropriate N applications such as ground water pollution and the release of nitrogen oxide greenhouse gases. Moreover, N fertilizers represent a significant part of the operational and energetic costs of wheat production. Most field genetic studies conducted at different N levels have reported significant genotype × N level and QTL × N level interactions for grain yield and grain protein concentration [83,84]. It seems, however, that very few have investigated the interaction between the genotype and the stress scenario. The BREEDWHEAT panel of elite Northern European winter varieties (BWP2 panel) was evaluated in 12 field trials with two N treatments. The clustering approach based on mean environment yield components and grain protein concentration identified four N availability scenarios: optimal condition, moderate and early deficiency, high and late deficiency, high and continuous deficiency (Agathe Mini, personal communication). A large range of tolerance to N deficiency was observed with varieties showing different rankings between N deficiency scenarios. The well-known negative correlation between grain yield and grain protein concentration also existed between tolerance indices calculated for these two traits, meaning that it will be difficult to identify varieties maintaining both their grain yield and protein concentration in N deficiency conditions. QTL regions were identified for the tolerance indices. These regions may be selected separately or combined thanks to their associated markers to improve the tolerance to N deficiency within a breeding program.

### 5.3. Crop Modeling

In the coming decades, plants are expected to be exposed to highly contrasting growth conditions and agricultural practices. In response to climatic and crop management changes, plants will shift their phenotype, which may affect their agronomic performances. Anticipating the plastic responses of plants is crucial to adapt their management and identify future varieties to be grown or bred. Given the large number of varieties and environments to be tested, computational plant modeling could help researchers and breeders identify efficient genotypes for given environments. Such plant models could build upon the large field trials and genotyping data that were recently collected in the BREEDWHEAT program [18,21], allowing to decompose the genotype-by-environment interactions (G × E).

Rincent et al. [61] proposed new methods to predict G × E interactions, which can help plant breeders to identify promising genotypes. To that purpose, the authors used a multi-environment trial involving 42 environments and 220 genotyped elite varieties of winter wheat (panel BWP2). The authors showed that an AMMI (additive main and multiplicative interaction) decomposition of the phenotypic data was very efficient to estimate observed covariances between varieties and between environments. In addition, two kinds of environmental covariates (EC) were used to characterize the environmental conditions: (i) usual meteorological data by developmental stage and (ii) stress indices derived from a crop model and reflecting the water, nitrogen, and temperature stresses that the plants might experience during their growth. Interestingly, they found that more G × E variance was explained when using a subset of seven ECs than with all ECs taken together (the correlation with the AMMI matrix increased from 0.56 to 0.84). The three most important ECs were the climatic variable related to the photo-thermal quotient between meiosis and flowering and two thermal stress indices depending on the maximal temperature during specific growth periods.

Using a similar approach, de los Campos et al. [85] presented a simulation platform to predict the performance of wheat cultivars in various weather conditions. This platform incorporates data from an extensive field trial on wheat (*n* = 25,841 records) including DNA polymorphism (SNPs), weather data, and EC generated from a crop model that reflects critical temperatures, radiation, and water availability for eight distinct phases of crop phenology. The authors used different statistical models to evaluate the proportion of grain yield variance explained by genetic and environmental factors and the fraction of those variances that could be captured using SNPs and ECs. They showed that most of the grain yield variance was explained by a so-called full model combining the trial information (year, location, and year × location interactions) along with ECs and SNPs. The molecular markers explained almost all of the genetic variance. In a cross-validation procedure, the full model also showed the highest within-trial correlation between predicted and observed grain yields (0.58). The authors then used the full model to predict the grain yield of 28 genotypes using historical weather data in 16 locations in France. Their simulations showed that modern cultivars achieved higher yields across locations than historical varieties. The simulation platform therefore offers an opportunity for researchers and breeders to leverage their data with historical weather records to produce robust predictions of cultivar performance.

Models of G × E interactions were also developed to optimize wheat phenology to avoid climatic stresses. Gouache et al. [86], and later Bogard et al. [87], used field trials involving a large number of winter wheat varieties grown in different French locations in order to calibrate a phenology model. In particular, two model parameters were calibrated using the information on molecular markers in Bogard et al. [87]. The two parameters GDDpv (growing degree days reduced by photoperiod and vernalization factors) and GDDp (growing degree days reduced by the photoperiod factor) determine the accumulation of modified thermal time by cold temperature and photoperiod from emergence to the beginning of stem elongation (GS30) and from GS30 to the heading date (GS55). Using a QTL-GBLUP (genomic best linear unbiased prediction) model in an independent dataset, the authors found that the correlation between predicted and observed GS30 and GS55 ranged from 0.37 to 0.71 and 0.74 to 0.94, respectively. GWAS for GDDpv and GDDp also showed the major impact of photoperiod sensitivity genes (Ppd-D1, Ppd-B1) on the earliness of the tested cultivars. Finally, simulations were performed for every marker-based GDDpv × GDDp combinations (*n* = 50,451), in order to identify the ideotypes maximizing climatic risk avoidance. They found strong interactions between the tested genotypes, sowing dates, and climate zones. For instance, early genotypes and late sowing should be preferred in oceanic regions with high Mediterranean influence (southwest of France). In contrast, the optimal growth period was overall shorter in semi-continental locations where early genotypes and early sowings should be avoided because of the high frequency of late frosts. The authors concluded that their methodology could be used to choose the genotype × sowing date combination that maximizes grain yield.

Barillot et al. [88] proposed a different and complementary approach that does not build on a statistical model of G × E, but rather on an explicit description of the processes involved in plant plasticity. The originality of the model, named CN-Wheat, lies in an integrated vision of plant functioning emerging from a detailed description of the primary metabolism of carbon (C) and nitrogen (N) at the organ scale. CN-Wheat is an individual-based model describing resource acquisition (photosynthesis, N-uptake, transpiration) as well as the synthesis, degradation, and allocation of the main C-N metabolites (sucrose, starch, fructans, nitrates, amino acids, and proteins). These physiological processes are not only regulated by pedo-climatic variables (light, CO_2_, moisture, and soil N), but also by metabolite concentrations. Thus, the metabolite concentrations at the organ level act as internal variables, allowing for the integration of the different processes at the scale of the whole plant. The model was first evaluated [89] for its ability to simulate the dynamics and spatial distribution of biomass and N between roots, photosynthetic organs, and grains, as observed in a field experiment where wheat plants were subjected to three levels of N fertilization at flowering. The model also provided clues for interpreting the observed behaviors, in particular, how the decrease in mobile metabolites following rapid grain filling, ultimately leads to the cessation of resource acquisition. The model was later used to assess how architectural traits such as leaf inclination affect resource acquisition and allocation in pure and mixed stands [90]. The model was recently extended to the vegetative stages [91], thus giving the opportunity to assess plant phenotypic plasticity in contrasting growth conditions [92].

### 5.4. High Throughput Field Phenotyping

While the genotyping capacity has increased rapidly, phenotyping has become the major limitation in research programs aiming at characterizing the genetic diversity for crop response to climate changes and reduced inputs [93]. Detailed measurements on a broad genetic diversity along the crop cycle and in known environmental conditions are key levers of genetic advances [94]. For that, both the development of platforms that enable creating environmental scenarios and monitoring plant development through sensors and models to derive relevant traits are necessary. While several traits can be well characterized under controlled conditions with plants in pots under greenhouses, emphasis should be put on field conditions that represent the actual challenge of phenotyping.

**Figure 4:**
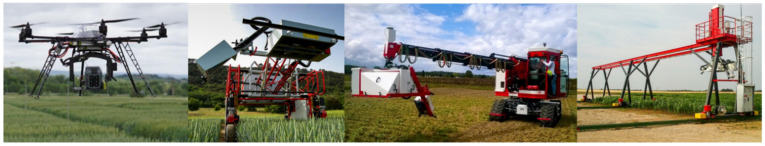
Different phenotyping systems used within the BREEDWHEAT project. From left to right: UAV equipped with a multispectral camera; Phenomobile V1 for crops lower than 1.2 m in height; Phenomobile V2 for crops up to 3 m in height; and the gantry system set up on PhenoField [95].

In close collaboration with the Phenome-Emphasis Project (https://www.phenome-emphasis.fr (accessed on 16 December 2021)), several phenotyping systems have been developed (Figure 4). These include UAV (unmanned airborne vehicles) equipped with high resolution RGB and multispectral cameras with throughput higher than 1000 micro-plots per hour (classical micro-plots are 7–12 m^2^ large). Furthermore, ground robotic rovers, called Phenomobiles, and gantry systems have also been specifically developed, with throughput higher than 100 micro-plots per hour. The Phenomobiles and gantry systems share the same measurement system, which includes LiDARs, RGB, and multispectral cameras. The sensors are triggered by the same unit that records, in a consistent way, all the data acquired along with the associated meta-information. The advantages and limits of the several phenotyping systems considered here are discussed below.

The phenotyping systems are operated over different installations including some (Ouzouer-le-Marché and Clermont-Ferrand) where the environment can be manipulated to control soil water availability with mobile rain shelters [95] and atmospheric CO_2_ concentration with a FACE (free air CO_2_ enrichment). Furthermore, the main environmental characteristics such as temperature, radiation, wind speed, air humidity, and soil moisture are precisely monitored at the hourly time step.

These high-throughput phenotyping systems deliver massive amount of data, mostly in the form of high resolution 2D and 3D images. A great effort was dedicated to developing interpretation methods that provide estimates of several relevant ecophysiological traits (Table 2). These correspond to structural, morphological, or biochemical characteristics either at the canopy or organ levels. The methods used are diverse, based on photogrammetry, the inversion of radiative transfer models, computer vision, and more recently, deep learning. Most methods use a single sensor, sometimes combining observations from two or more directions to better scan plants or organs and better describe the canopy structure. However, for canopy level traits, several methods and sensors can be combined, resulting in multiple estimates that can be used to check the consistency of high-throughput methods. While the traits derived from the LiDAR are specific to the Phenomobile, most of the other traits can be derived either from UAV or Phenomobile observations. The higher throughput of UAV allows for exhaustive sampling of the micro-plots, although this is obtained at the expense of the quality of the images with a reduced ground sampling distance. Conversely, Phenomobile observations are lower throughput, but allow for the control of the illumination conditions by using flashes, and to obtain a higher spatial resolution (smaller ground sampling distance) because of the slower speed of the vehicle, and smaller distance between the sensors and the ground. A very high spatial resolution is mandatory when estimating organ level traits because they first need to be identified, requiring a ground sampling distance at least smaller than one fifth the typical organ dimension. Deep learning methods based on convolutional neural networks are now very efficient for segmentation and plant or organ identification and counting. They are currently assessed to score symptoms of diseases targeting specific organs. However, the main drawback of such machine learning methods is the difficulty in obtaining a robust model. It therefore requires a large and diverse training dataset populated by many acquisition sessions with possible differences in ground sampling distance, illumination conditions, growth stages, and background. However, for most of the traits considered in Table 2, the methods that we developed showed a higher broad sense heritability compared to the traditional phenotyping methods.

The high throughput phenotyping methods targeting traits describing canopy or the organ state allowed us to repeat the observations along the growth cycle. It is then possible to access a few phenological events such as heading [110] or flowering [97], and to describe the dynamics of canopy structure as a proxy of functional traits. The use of simple models or more sophisticated ones [101,107,111,112] offers great potential for providing breeders with new insights into the functioning of the crop. This will be the focus of future investigations where crop functioning models are combined with high-throughput phenotyping observations to tune model parameters that describe the reaction of the crop to environmental factors. This will ultimately allow us to predict the fate of the crop for a wide range of environmental conditions.

## 6. Development of Innovative Methods and Cost-Efficient Breeding Platforms

### 6.1. Genomic Selection

As stated previously, accelerating genetic progress has become the priority of many agricultural agencies. Genetic progress per year (∆G) is given by the general formula:

∆G = (i × h^2^ × σ_ρ_)/L

where i is the selection intensity; h^2^ is the trait heritability; σ_ρ_ is the phenotypic variability; and L is the duration of the selection cycle. The utilization of genome-wide markers has been proposed as a means to increase selection intensity and reduce cycle length (by reducing the need/cost of phenotyping), provided that marker-based prediction accuracy is compared to phenotypic heritability. This was proposed as “genomic selection” (GS) by Meuwissen et al. [113] and has since been widely applied, particularly in animal genetics for quantitative traits such as milk production controlled by “infinitesimal” genes. The use of GS for crop improvement was first proposed in 2007 by Bernardo and Yu [114]. However, in contrast to animals, plants are subjected to huge G × E interactions. In addition, in the case of wheat, breeding objectives are diverse and encompass complex traits such as yield under stress conditions, and more simple traits such as quality or disease resistance. In 2011, only three papers dealing with GS in wheat were published [115,116,117]. Later, several genomic prediction models have been applied to various traits of bread wheat [118]. Although differences were usually small, some methods achieve higher predictive abilities than others, depending on the traits considered, most likely due to their genetic architecture.

**Figure 5:**
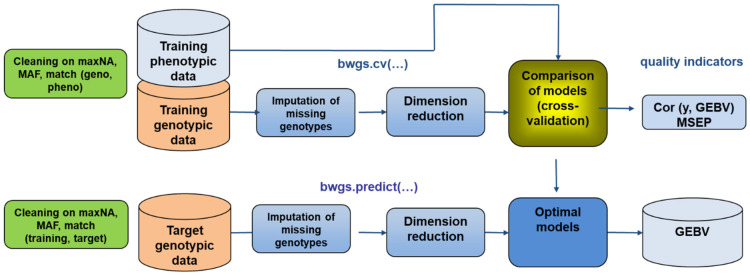
Workflow of the two main functions of the BREEDWHEAT Genomic Selection (BWGS) software. Function bwgs.cv() derives model cross-validation on a training set and function bwgs.predict() conducts model calibration on a training set and GEBV prediction of a target set of genotypes. MAF: minor allele frequency, maxNA: maximum % of marker missing data, MSEP: mean square error of prediction, GEBV: genomic estimated breeding value.

**Figure 6:**
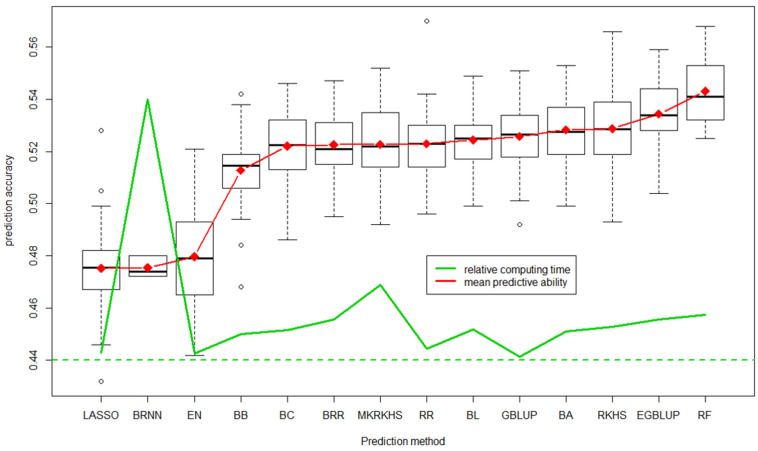
Distribution of predictive ability of the 100 replicates for each of the 14 methods ordered based on average predictive accuracy [120]. Average is shown in red and relative computing time in green. LASSO: least absolute shrinkage and selection operator, BRNN: Bayesian regularization for feed-forward neural network, EN: elastic net, BB: Bayes B, BC: Bayes C, BRR: Bayesian ridge regression, MRKHS: multiple RKHS, RR: ridge regression, BL: Bayesian LASSO, GBLUP: genomic best linear unbiased prediction, BA: Bayes A, RKHS: reproductive Kernel Hilbert space, EGBLUP: epistatic GBLUP, RF: random forest. The box are interquartile limits, dashed traits the 95% distribution and ° the outliers.

The first task in BREEDWHEAT was to develop an integrated pipeline for genomic prediction based on available R-libraries [119]. The BREEDWHEAT Genomic Selection software, BWGS [120] (Figure 5), is available as an easy-to-use, standalone R package, with default parameters adapted to the commonly used size of datasets. It includes two methods for missing data imputation, four methods for selecting marker subsets (random, LD-based, GWAS-based), and 14 methods for GEBV prediction (from historical GBLUP to Bayesian radial neural networks). Additionally, two methods are proposed for sampling training subsets: random (useful for teaching purpose) and optimization, according to Rincent et al. [121]. A first function enables carrying out random cross-validation to select the best parameterization and the most predictive method, and a second function to carry out the prediction of GEBV in the target set of lines after designing the best model on the training set. The source code of BWGS R functions as well as example files and notes are freely available on https://forgemia.inra.fr/umr-gdec/bwgs (accessed on 16 December 2021), and also from the CRAN (https://cran.r-project.org/ (accessed on 16 December 2021)). Figure 6 illustrates the predictive ability of the 14 prediction methods obtained for grain yield by cross-validation on a training population of 760 breeding lines with historical trial data from 2000 to 2014 (unbalanced data).

BWGS was primarily developed to estimate single trait GEBV. However, wheat breeding requires simultaneous improvement of several traits, which are often correlated to each other. This is why we also tested methods for multi-trait genomic selection and trait-assisted genomic selection [122]. In this study, an extension of the optimization algorithm from Rincent et al. [121] was proposed, and a case study was carried out on bread making quality using data from a real breeding program. Results were presented considering the respective cost of genotyping and phenotyping for both the main and the secondary traits.

Such economic parameterization was also used in a virtual selection program using a novel algorithm, whose parameters were co-constructed with BREEDWHEAT breeders. This software enables the breeder to compare realistic breeding schemes of similar cost, with or without a step of genomic pre-selection [123,124].

We also addressed the endless question of predicting G × E interaction. Although several attempts have been published thus far [118,125], the improvement provided by these methods appears to be limited. We used genomic random factorial regression (FR-GBLUP) to achieve genome prediction of the reaction norms to environmental stress [126]. This tool can help breeders to improve adaptation and tolerance to specific stress factors such as heat or drought. Comparing the prediction accuracies of the additive GBLUP and the FR-GBLUP models on real data, we showed accuracy gains of 1.6 to 26.2% for the genomic regression to drought. To predict performances of individuals in new environments, the FR-GBLUP model was consistently more accurate than the additive GBLUP. Moreover, we reported that the use of output variables from crop growth models (CGM) allowed higher predictive ability of FR-GBLUP than raw variables (e.g., climatic), as exemplified with the use of the nitrogen nutrition index on grain number [127]. In addition, a new prediction approach combining the use of a secondary trait and a CGM was proposed [19]. The originality is that the phenotyping of the test set for the secondary trait is replaced by CGM predictions. Prediction of grain yield as the target trait using heading date as the secondary trait resulted in high predictive abilities in three prediction scenarios (sparse testing, or prediction of new genotypes, or of new environments).

Finally, we wanted to use CGM incorporating genetic parameters to combine ecophysiological and genetic modeling. The objective was to determine a method to optimize the set of environments composing a multi-environment trial (MET) for estimating genetic parameters. A criterion called OptiMET was defined for this aim, and was evaluated on simulated and real data, with the example of wheat phenology. The MET defined with OptiMET allowed us to estimate the genetic parameters with lower error, leading to higher QTL detection power and higher prediction accuracies. MET defined with OptiMET was on average more efficient than random MET composed of twice as many environments in terms of genetic parameters estimation [128].

### 6.2. Phenomic Selection

Obtaining high-density genotyping data on large numbers of individuals such as those that are typically experienced in breeding can be challenging and expensive. In 2009, Mackay et al. [129] proposed the use of endophenotypes (i.e., intermediate molecular phenotypes such as transcripts, proteins or metabolites) to help accurately predict complex traits. The possibility of using metabolic markers for monitoring or predicting plant performance was reviewed by Fernandez et al. [130]. A cost-efficient pipeline using metabolic markers as putative predictors of performance with notable applications in plant-breeding was designed. In this context, Rincent et al. [18] proposed using low-cost and non-destructive near-infrared spectroscopy (NIRS) to perform “phenomic selection” (PS) based on high-throughput phenotyping to obtain numerous variables that can be used as regressors or to estimate kinship in the classical statistical GS models. In PS, NIR reflectances are considered in the same way as genomic or endophenotypic regressors, allowing for predictions in any environment once NIR reflectances are acquired in one environment. Tested on bread wheat, predictions as accurate as those with molecular markers were reached for heading date and grain yield, even in environments radically different from the one in which NIRS were collected. These studies constitute a proof of concept and provide new perspectives for the breeding community [131].

## 7. Data Integration into an Information System following the FAIR Principles

The BREEDWHEAT data are very valuable, diverse, and high throughput. To manage these data, we used the GnpIS information system of INRAE-URGI, which allowed us to achieve important objectives:

(1) Long-term storage of the data as GnpIS has been available since 2000 and benefits from perennial funding by INRAE (Plant Biology and Breeding division);
(2) Implementation of a data management plan, which includes a data access mechanism with credentials following the consortium agreement;
(3) Integration of all the project data in a common information system to link the data from genomics to phenomics [132];
(4) To allow the researchers and breeders to query the data through the GnpIS web interfaces (FAIDARE, JBrowse, GnpIS core-DB, detailed below); and
(5) To insure data quality and compliance to the FAIR principles (Findable Accessible Interoperable Reusable) [133].

**Figure 7:**
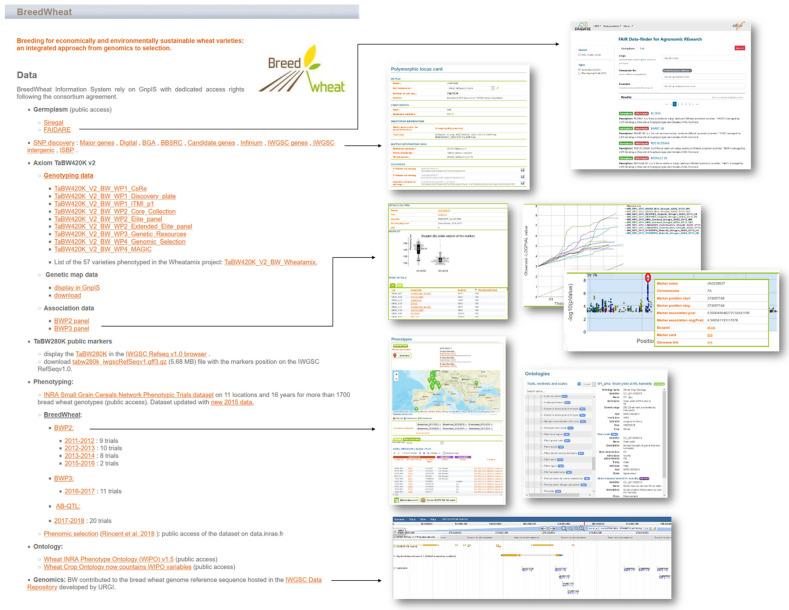
BREEDWHEAT data summary webpage (https://wheat-urgi.versailles.inrae.fr/Projects/BreedWheat (accessed on 16 December 2021)) with some examples of GnpIS web interface results.

A dedicated webpage has been set up to easily access the BREEDWHEAT data in GnpIS: https://wheat-urgi.versailles.inrae.fr/Projects/BreedWheat (accessed on 16 December 2021) (Figure 7).

### 7.1. Data Quality and FAIRness

The project data followed the existing standards per data type (e.g., VCF, MIAPPE, MCPD) and have been curated. Moreover, consistency checks have been performed using ETL (Extract Transform Load) tools. In addition to these quality checks, GnpIS insures the FAIRness of the data and metadata:

- Findability: a DOI (digital object identifier) was generated for each accession; all data are searchable using web interfaces; public BREEDWHEAT data are findable by the whole community via the WheatIS data discovery tool (https://urgi.versailles.inrae.fr/wheatis (accessed on 16 December 2021)).
- Accessibility: phenotyping data are accessible through Breeding API (BrAPI) web services [134].
- Interoperability: phenotyping data followed an ontology developed in the frame of the project and merged with the international wheat crop ontology (CO_321) [135].
- Reusability: a data management timeline defines when each kind of data will be opened; all the GnpIS tools have general terms of use and license.

### 7.2. Genetic Resources Data Integration

The data of 5232 accessions managed by the INRAE Biological Resource Center were integrated in GnpIS. The passport data and the related phenotyping data can be displayed using the FAIDARE (FAIr Data-finder for Agricultural REsearch) web interface. FAIDARE allows one to find data using free text, controlled vocabularies, and identifiers of key scientific resources such as genetic material or phenotyping and environmental traits. It indexes databases around the globe and offers data preview of all those datasets with relevant ontology annotation when available with a link back to the original database.

### 7.3. Genomics Data Integration

The 1B chromosome sequence obtained in the frame of BREEDWHEAT was included in the reference sequence of the IWGSC available in the IWGSC data repository hosted by INRAE-URGI. GnpIS offers some tools to download, analyze (BLAST), and display (JBrowse) this annotated genome. The IWGSC RefSeq v1.0 browser includes a dedicated track to display the TaBW280K SNPs chip [14]. All the annotated markers including SNPs were linked to the corresponding genetic and phenomic data [132].

### 7.4. Genotyping, Phenotyping, and GWAS Data Integration

GnpIS core-DB is a hybrid database composed by a relational database (PostgreSQL) that allows for a high level of data integration though well curated pivot data and a NoSQL database (ElasticSearch), which enables fast searchable capabilities. All the SNPs of the Axiom TaBW410K chip (including the TaBW280K first published markers) [14] and the corresponding genetic map were integrated in GnpIS. SNP positions on the reference genome and their flanking sequences are available as well as a custom download in a VCF format. Phenotyping data from panel BWP2 (29 trials since 2011), panel BWP3 (11 trials since 2016), and AB-QTL (20 trials since 2017) were integrated in GnpIS following the same trait ontology [136], which allows one to search for a variable across all of the BREEDWHEAT phenotyping experiments with download capabilities in standard formats such as MIAPPE and ISA-TAB.

GnpIS integrated the corresponding GWAS between the BREEDWHEAT genotyping and phenotyping data. It also offers figures displayed as box plots, Manhattan plots, and QQ plots. It integrated the BREEDWHEAT data together, but also with open access data from the achieved project (e.g., H2020 Whealbi, www.whealbi.eu (accessed on 16 December 2021)), public networks (e.g., French small grain cereals network), and consortia (e.g., IWGSC, Wheat Initiative). This will help researchers and breeders find new knowledge.

## 8. Conclusions

The BREEDWHEAT project was conceived to provide wheat breeders with extension services with knowledge and tools to address the societal demand for wheat production sustainability, quality, and safety. It contributed significantly to the sequencing and the analysis of the first reference sequence of the hexaploid wheat genome, which was a major breakthrough for the wheat community. Based on these resources, two genotyping arrays were developed: the TaBW410K and the TaBW35K arrays, which were extensively used in the framework of BREEDWHEAT as well as in other projects, since they have been made available to the wheat community. More than 13,000 cultivated and wild wheat accessions were genotyped to perform genome-wide association studies, implement genomic and phenomic selection, characterize the worldwide diversity and its history, and build a catalogue of structural variations of the genome sequence.

The aim was also to broaden the genetic basis of French elite varieties through the introduction of novel sources of genetic diversity that contain favorable alleles for resistance to abiotic and biotic stresses. For this, plans were made (i) to select a panel of 450 accessions representing the world genetic diversity for winter wheat and (ii) to create pre-breeding populations crossing elite European varieties and exotic genitors, bringing new tolerances to biotic and abiotic stresses. To build the panel, 4600 accessions of the INRAE bread wheat collection were genotyped and characterized in the field for adaptation traits (plant height, precocity). A total of 450 accessions were then selected in order to maximize the power of detecting chromosomal regions in genome-wide association studies. To create pre-breeding populations, genitors were either selected in the worldwide diversity or in the panel of 450 accessions. Once these selections were made, BREEDWHEAT partners created 36 populations for a total of almost 5000 pre-breeding lines that contain novel sources of diversity for tolerance to various stresses.

The genetic and ecophysiological tolerance to biotic and abiotic stresses were investigated. A set of ecophysiological models was developed to investigate genotype × environment interactions and improve methods for high throughput phenotyping. Candidate genes and pathways were identified for the response of grain weight to high temperature and the response of grain protein composition to nitrogen and sulfur supplies. This was undertaken using different approaches (network inference, multivariate analyses) to analyze transcriptomics and proteomics data. Finally, genetic approaches were undertaken to identify genomic regions of interest (QTL) by combining genotypic and phenotypic data collected from large field trial networks. Two different panels, the first one composed of 220 European elite varieties and the second one corresponding to the 450-accessions worldwide diversity panel, were experimented and phenotyped in large trial networks. Marker x traits associations were identified for tolerance to stresses. BREEDWHEAT was able to provide lists of tolerant varieties and markers to breeders in order to follow genetic areas of interest for biotic and abiotic stress tolerance.

An R-package named BWGS, which gathers several easy-to-use methods for data cleaning, sampling, and imputation as well as 15 methods for genomic prediction of breeding value was developed. This package was disseminated through national and international training sessions. The objective was also to develop a centralized repository suited to the data generated within BREEDWHEAT and following the FAIR principles. It was specifically designed to manage and link different data types: genomics data, germplasm description, genotyping data, genetic maps, ontologies, phenotyping data, and the results of association studies. The information system was successfully implemented with the data and a dedicated webpage was set up to easily access them.

Further works on traits of interest are needed in many areas. Recent future climatic scenarios [137] do not predict a change in the trend of increased constraints for crops cultivated in Northern Europe. Average temperature increase will lead to accelerated development rates and shorter growth cycle. Even in the case of no change in the amount of rainfall, less water will be available due to higher evapotranspiration. Furthermore, extreme events are likely to be more frequent such as what happened for the 2015–2016 season in France with a combination of abnormally warm temperatures in late autumn 2015 and abnormally wet conditions and low solar radiations in spring 2016 [138]. In addition, economic and ecological constraints will favor agricultural systems with less phytosanitary and fertilizer inputs such as those prone in organic farming and agroecology [139]. Favorable biotic interactions (e.g., plant-to-plant interactions in cultivar or species mixtures and plant–microbiome interactions in the rhizosphere) will probably be important factors to design less vulnerable and more resilient agricultural systems. In these conditions, maintaining the genetic progress will be a huge challenge. This will rely on the optimal use of all recent breeding tools (high-throughput genotyping and phenotyping, genomic and phenomic selection) [140,141] and the exploitation of all available genetic diversity that could best be conducted within large consortia associating public and private research.

## Figures and Tables

**Table 1:** Summary of genomics tools developed within the BREEDWHEAT project.

Tool	Size	Uses	Publications
Axiom SNP arrays	409,685 SNPs	Phylogeny, mapping, GWAS, GS	[14,15,16,17,18,19,20,21]
	34,746 SNPs	GWAS, GS	
Chinese Spring (CS) × Renan Genetic map	146,602 SNPs	21 CS pseudomolecules assembly, analysis of the recombination landscape, QTL detection	[22,23,24,25,26]
Chromosome 1B sequence	10,395 BACs 13,277 scaffolds 920 Mb	Analysis of the transcriptional landscape, the impact of transposable elements on genome structure and evolution, etc.	[22,23,24,25]

**Table 2:** Traits that can be estimated from high-throughput systems developed within BREEDWHEAT and Phenome-Emphasis projects that can be applied to wheat crops. DL: deep learning, RTM: radiative transfer model, SVM: support vector machine, VI: vegetation index. Colored cell indicate that the corresponding sensor or vector was used to estimate the trait.

Level	Trait	Method	Sensor			Configuration		Vector		Reference
			RGB	Multispectral	LiDAR	View Direction	Ground Sampling Distance (cm)	UAV	Phenomobile	
Canopy	Vegetation Index (VI)	Band combination				0°	20			[96]
	Plant height	Structure from motion				0°	1			[97]
		Distribution of height				±35°	0.5			[97]
	Vegetation Fraction (VF)	DL segmentation				0°	0.05			Madec et al. (pers.com)
		Height threshold				±35°	0.5			Lopez-Lozano et al. (pers.com)
	Green Fraction (GF)	SVM/random forest				0°-45°	0.05			Serouart et al. (pers.com)
		DL segmentation				0°–45°	0.05			Madec et al. (pers.com)
		1D RTM inversion				0°	20			[98]
	Green Area Index (GAI)	Green fraction turbid				0°–45°	0.05			[99]
		1D RTM inversion				0°–45°	20			[100]
		3D RTM inversion				±35°	0.05–0.5			[101]
	Plant Area Index (PAI)	1D turbid				±35°	0.5			Lopez-Lozano et al. (pers.com)
	Fraction of Intercepted Radiation (FIPAR)	1D RTM inversion				0°	20			[102]
		Green Fraction turbid				0°–45°	0.05			[103]
		1D turbid				±35°	0.5			Lopez-Lozano et al. (pers.com)
	Average Inclination Angle (AIA)	1D RTM inversion				0°	20			[102]
		1D turbid				0°–45°	0.05			Liu et al. (pers.com)
		1D turbid				±35°	0.5			Lopez-Lozano et al. (pers.com)
		3D inversion				±35°	0.05–0.5			[103]
	Canopy Chlorophyll Content (CCC)	1D RTM inversion				0°–45°	20			[104]
		VI empirical				0°	20			[105]
		VI empirical				0°	0.05			[106]
	3D Distribution of Area	1D turbid				±35°	0.5			Lopez-Lozano et al. (pers.com)
Organ	Plant density	DL at emergence				45°	0.05			[107]
	Stem density	DL at harvest				0°	0.02			[108]
	Stem diameter	DL at harvest				0°	0.02			[108]
	Ear density	DL at reproductive stage				0°	0.05			[109]
	Leaf Chlorophyll Content	1D RTM inversion				0°	20			[105]
		VI empirical				0°	0.05			Jay et al. (pers.com)
		VI empirical				0°	0.05			Jay et al. (pers.com)

## Data Availability

Data in GnpIS: https://wheat-urgi.versailles.inrae.fr/Projects/BreedWheat (accessed on 1 December 2021)) are freely available.
